# Childbearing during adolescence and offspring mortality: findings from three population-based cohorts in southern Brazil

**DOI:** 10.1186/1471-2458-11-781

**Published:** 2011-10-10

**Authors:** María C Restrepo-Méndez, Aluísio JD Barros, Iná S Santos, Ana MB Menezes, Alicia Matijasevich, Fernando C Barros, Cesar G Victora

**Affiliations:** 1 Postgraduate Programme in Epidemiology, Federal University of Pelotas, Rua Marechal Deodoro, 1160 3° Piso, 96020-220, Pelotas, Brazil; 2 Postgraduate Course in Health and Behavior, Universidade Católica de Pelotas, Rua Félix da Cunha, 412 - 96010-000, Pelotas, Brazil

**Keywords:** pregnancy in adolescence, infant mortality, neonatal mortality, perinatal mortality, fetal mortality, cohort studies

## Abstract

**Background:**

The role of young maternal age as a determinant of adverse child health outcomes is controversial, with existing studies providing conflicting results. This work assessed the association between adolescent childbearing and early offspring mortality in three birth cohort studies from the city of Pelotas in Southern Brazil.

**Methods:**

All hospital births from 1982 (6,011), 1993 (5,304), and 2004 (4,287) were identified and these infants were followed up. Deaths were monitored through vital registration, visits to hospitals and cemeteries. The analyses were restricted to women younger than 30 years who delivered singletons (72%, 70% and 67% of the original cohorts, respectively). Maternal age was categorized into three groups (< 16, 16-19, and 20-29 years). Further analyses compared mothers aged 12-19 and 20-29 years. The outcome variables included fetal, perinatal, neonatal, postneonatal and infant mortality. Crude and adjusted odds ratios (ORs) were estimated with logistic regression models.

**Results:**

There were no interactions between maternal age and cohort year. After adjustment for confounding, pooled ORs for mothers aged 12-19 years were 0.6 (95% CI = 0.4; 1.0) for fetal death, 0.9 (0.6; 1.3) for perinatal death, 1.0 (0.7; 1.6) for early neonatal death, 1.6 (0.7; 3.4) for late neonatal death, 1.8 (1.1; 2.9) for postneonatal death, and 1.6 (1.2; 2.1) for infant death, when compared to mothers aged 20-29 years. Further adjustment for mediating variables led to the disappearance of the excess of postneonatal mortality. The number of mothers younger than 16 years was not sufficient for most analyses.

**Conclusion:**

The slightly increased odds of postneonatal mortality among children of adolescent mothers suggest that social and environmental factors may be more important than maternal biologic immaturity.

## Background

Adolescent childbearing has received wide attention in public health. From 2005-2010, age-specific fertility rate among women aged 15-19 years old were 21 live births per 1,000 women in high-income, 47 in middle-income and 103 in low-income countries [1]. In Brazil, adolescent pregnancies account for one in every five live births [2]. Nevertheless, the age-specific fertility rate among Brazilian women aged 15-19 years old has declined from 84 live births per 1,000 women in 1996 to 77 in 2007 [3]. Infant mortality is also declining rapidly in Brazil, from 47 deaths per 1,000 live births in 1990 to 20 in 2007 [4], but it still remains higher than most large countries in Latin America [5]. Data from vital statistics, which covers about 72% of infant deaths in the country [6], shows that infant mortality rate ratios were 2.2 for mothers aged 10-14 years and 1.3 for 15-19-year-olds, compared to those aged 20-29 years [2,7]. These results are not adjusted for confounding variables, since the necessary information for adjustment is not available in the records, or is not reliable.

Globally, substantial literature can be found to either support or refute the role of low maternal age as a determinant of adverse offspring outcomes, which include low birth weight [8-18], preterm birth [8-13,15,18,19], intrauterine growth restriction [9-11,13], neonatal and infant mortality [9-13,15-18,20-24]. Some studies suggest that biological immaturity due to young age increases the risk of these outcomes. Other authors argue that most reported associations are due to confounding by social and environmental factors, as in most societies adolescent childbearing is associated with low socioeconomic status, poor education, unmarried status, minority ethnic group affiliation, and inadequate prenatal care [9,13,25].

Research regarding the effects of adolescent childbearing on offspring outcomes may be affected by methodological limitations. Most studies used secondary data from clinical or vital registration systems, and many lack information on potential confounders [12,18,23,26]. Some findings were based on small sample sizes, mainly for very young mothers (< 15 years) [14,27]. Many studies fail to adjust for known confounders or to consider effect modification, which may lead to overestimation of the effect [12,18,23,26,28-30]. Others treat mediating factors as confounders, and therefore may underestimate existing associations [15,19,22]. In addition, the reference group often includes mothers aged 30 years or more who are also at increased risk of some outcomes, and this might conceal the effect of young maternal age on offspring outcomes [14,17,19,22]. Finally, some of the analyses fail to take into account the differences in biological and psychological maturity between younger and older adolescents [14,17,19,24].

In order to assess the possible association between adolescent childbearing and increased risk of offspring mortality, data from three large population-based birth cohort studies were compared. These studies, which were carried out in a region with large social inequalities, provide a rich source of data for exploring this association while overcoming some of the limitations of earlier studies. They also allow examination of whether the relationship between maternal age and child health is changing over time.

## Methods

Pelotas is a city located in southern Brazil with nearly 330,000 inhabitants. More than 98% of deliveries take place in hospitals. In 1982, 1993 and 2004, three similar perinatal studies were conducted including all hospital births in the city (6,011, 5,304 and 4,287 total births, respectively). Mothers were interviewed soon after delivery using a standardized questionnaire and provided information regarding socioeconomic and demographic characteristics, reproductive health and healthcare during pregnancy and delivery. In addition, children and their mothers were weighed and measured with calibrated equipment. Subsequent follow-ups were carried out in all three cohorts with some small methodological differences. More details about the methods employed in these three studies are described elsewhere [31-33].

The present study was restricted to singleton births of mothers younger than 30 years. Because mothers older than 30 years were regarded as a potentially higher-risk group for adverse birth outcomes, they were excluded from analyses. Therefore the study sample was limited to 72%, 70% and 67% from the original cohorts, respectively.

The outcome variables included fetal, perinatal, neonatal, postneonatal and infant mortality rates. Deaths were monitored through regular visits to maternity wards and to intensive and intermediate care centers. Further regular visits were made to cemeteries, to the local vital registration offices and to the Regional and Municipal Health Secretariat in order to track deaths which took place outside hospitals. Detailed descriptions of the methods used for ascertaining mortality are available [34,35]. In 1982, the definition used for fetal death was a death occurring after the 28^th ^week of gestation or fetuses larger than 1,000 g when gestational age was unknown. This definition was also applied in 1993 and 2004 in order to compare the three cohorts, even though the definition of fetal deaths was changed in 1993 [36]. The definition used in the present analyses refers to what is currently known as late fetal deaths. Perinatal deaths equal the sum of late fetal deaths and early neonatal deaths (live-born children who died 0-6 days after birth). Infant deaths referred to live-born infants who died throughout the first year of life (0-364 days). Late neonatal (7-27 days) and postneonatal mortality (28-364 days) were also studied. Fetal and perinatal mortality rates were both expressed as the number of deaths per 1 000 total births (stillbirths and live births). Neonatal, postneonatal and infant mortality rates were denoted as the number of deaths per 1 000 live births.

Maternal age at the time of delivery was categorized into three groups (< 16, 16-19, and 20-29 completed years). Whenever possible, the two subgroups of adolescent mothers (< 16 and 16-19) were compared to mothers aged 20-29 years, who constituted the reference category because they were expected to have the lowest risk of adverse pregnancy outcomes. When sample sizes did not allow disaggregation into three age groups, adolescent mothers as a whole (aged 19 years or less) were compared to the reference group.

Potential confounding variables considered for adjustment included family income, maternal education, maternal skin color, marital status, parity and pre-pregnancy body mass index (BMI). Those variables were collected during the perinatal studies, except for maternal height (used to calculate pre-pregnancy BMI) that was measured at the three-month follow-up in the 2004 cohort study. The same variable definitions were applied in all three studies. Family income was expressed in minimum wage per month (a minimum wage was worth about US$50 in 1982, US$60 in 1993, and US$80 in 2004). Maternal education was defined as number of completed years of schooling. Maternal skin color was classified by the interviewers and categorized as white or black/mixed. In characterizing marital status, women who had a live-in partner were treated as married, regardless of their official civil status. Parity was determined by the number of previous births, including stillbirths. Pre-pregnancy weight was gathered from prenatal records at the woman's first antenatal care or by maternal recall at the time of delivery in case the data were missing from records. Pre-pregnancy BMI was calculated by weight divided by height squared, as measured by the study team (kg/m^2^).

Additional adjustment for potential mediating variables was also considered when a significant association was found after controlling for confounders. Three groups of mediators were considered: variables relating to pregnancy and delivery (maternal weight gain, number of antenatal visits, smoking, complications such as hypertension, diabetes and threatened abortion, and type of delivery), to the newborn (gestational age at delivery and birth weight) and breastfeeding practices (duration of total breastfeeding). Maternal weight gain during pregnancy was based on the difference between initial and final weight. In the 1982 and 1993 cohorts, final weight was measured in the hospital upon admission. In 2004, this weight was transcribed from the mother's card. The number of prenatal care attendances was taken from existing records or, if unavailable, obtained through maternal self-report. Maternal smoking was defined as the consumption of at least one cigarette per day, in any of the trimesters of pregnancy. Presence of morbidity during pregnancy - such as hypertension, diabetes and threatened abortion - and type of delivery was reported by the mothers in the hospital interview. Gestational age in completed weeks was defined as the interval between the first day of the last normal menstrual period and the date of birth. Birth weight was determined using the hospital scales, which were regularly calibrated by researchers. Breastfeeding data was collected at different follow ups from 1 to 48 months.

Chi-squared tests (χ^2^) were used to determine differences in socio-demographic and reproductive health characteristics across the groups of maternal age for each cohort study. Chi-squared tests for linear trend were also performed when appropriate. The effect of maternal age was initially assessed using each mortality outcome in separate analyses, in one cohort at a time. Crude and adjusted odds ratios (ORs) and 95% confidence intervals (95% CI) were estimated using logistic regression models. Potential confounders were included in backward selection regressions, and those that remained associated (p < 0.2) with both maternal age and mortality outcomes in at least one of the three cohorts, were retained. All potential confounders remained in the model, except for pre-pregnancy BMI. Interactions between maternal age and cohort year were evaluated by testing product terms in adjusted models. Pooled effect estimates from the combination of three cohort samples were also calculated whenever interaction terms had a p level > 0.2. In these cases, both crude and adjusted ORs were additionally controlled for cohort year. Further interaction terms were explored between maternal age and family income, marital status, mother skin color, or children's sex respectively. Since none of these terms reached a p level < 0.2, stratified analyses were not performed. The Hosmer-Lemeshow goodness of fit test was applied to examine adequacy of final models fit. All analyses were performed using the Stata Statistical Software, version 11.0 (Stata Corp., College Station, USA).

The study protocol was approved by the Medical Ethics Committee of the Federal University of Pelotas. In 1982 and 1993 verbal consent to participate in the studies were obtained from mothers and written consent was also requested in 2004.

## Results

The percentages of all singleton infants born to mothers aged 12 to 19 years were 15.5%, 17.6% and 19.1% in 1982, 1993 and 2004 respectively, and fewer than 3% of babies were born to mothers aged 12 to 15 years in each cohort (Table 1). However, the absolute number of mothers aged 12 to 15 years increased by 75% from 1982 to 2004. The age-specific fertility rates among women aged 15-19 years old were 83 live births per 1,000 women in 1982, 73 in 1993 and 54 in 2004. For girls aged 10-14 years, the corresponding rates were 1.4, 2.3 and 2.9 live births per 1,000, respectively.

**Table 1:** Distribution of maternal age among singleton births, by cohort study

	1982	1993	2004	Pooled cohorts
	**N (%)**	**N (%)**	**N (%)**	**N (%)**

**Maternal age, y**				
12-15	65 (1.1)	108 (2.1)	114 (2.7)	287 (1.9)
16-17	296 (5.0)	325 (6.2)	289 (6.9)	910 (5.9)
18-19	556 (9.4)	485 (9.3)	400 (9.5)	1,441 (9.4)
20-29	3,424 (58.0)	2,779 (53.2)	2,084 (49.6)	8,287 (54.1)
30-39	1,424 (24.1)	1,406 (26.9)	1,172 (27.9)	4,002 (26.1)
40-49	143 (2.4)	119 (2.3)	140 (3.3)	402 (2.6)
**Total**	5,908 (100)	5,222 (100)	4,199 (100)	15,329 (100)
**Number of mothers less than 30 y**	4,341	3,697	2,887	10,925

Pelotas, Brazil, 1982, 1993, and 2004

Perinatal mortality rates dropped from 27.3 per 1,000 births in 1982 to 18.8 in 2004 among adolescent mothers, and from 23.4 in 1982 to 17.8 in 2004 among 20-29-year-old mothers. Infant mortality rates also declined from 47.4 per 1,000 live births in 1982 to 29 in 2004 among adolescent mothers, and from 32.2 to 19.4 among 20-29-year-old mothers.

Over the two decades, several changes occurred in terms of maternal characteristics (Table 2). In the three cohorts, adolescents had lower family income, lower BMI, lower parity and were less likely to live with a partner, compared to mothers aged 20-29 years, but there were no differences in terms of skin color. In 1982 and 1993, adolescents also had lower schooling, but by 2004 this trend had reversed.

**Table 2:** Characteristics of mothers with singleton births, by maternal age

	1982			1993			2004		
	
Variables	< 16	16-19	20-29	< 16	16-19	20-29	< 16	16-19	20-29
	%	%	%	%	%	%	%	%	%
**Family income (minimum wage)**									
≤ 1.0	46.9	36.0	20.7	27.8	23.5	19.5	36.8	30.8	20.5
1.1 -3.0	50.0	52.5	48.1	48.2	48.4	43.3	50.9	50.4	47.1
3.1 - 6.0	3.1	8.4	19.7	17.6	21.1	23.6	10.5	15.3	23.5
> 6.0	0.0	3.1	11.5	6.5	7.0	13.7	1.8	3.5	8.8
**Total (N)**	64	844	3,409	108	810	2,779	114	688	2,081
P-value	< 0.001^a^			< 0.001^a^			< 0.001^a^		
	< 0.001 ^b^			< 0.001 ^b^			< 0.001 ^b^		
**Schooling (years)**									
0	6.2	4.6	3.6	5.6	2.1	2.4	0.0	0.3	0.9
1 - 4	47.7	35.2	25.5	31.5	27.9	26.0	16.7	12.3	12.9
5 - 8	41.5	50.8	42.9	61.1	58.4	45.8	75.4	61.1	36.9
9 +	4.6	9.4	28.0	1.9	11.6	25.8	7.9	26.3	49.3
**Total (N)**	65	852	3,419	108	810	2,776	114	689	2,072
P-value	< 0.001^a^			< 0.001^a^			< 0.001^a^		
	< 0.001 ^b^			< 0.001 ^b^			< 0.001 ^b^		
**Skin color**									
White	78.5	80.1	82.7	77.8	72.6	77.6	70.2	72.7	72.9
Black and Mixed-race	21.5	20.0	17.3	22.2	27.4	22.4	29.8	27.3	27.1
**Total (N)**	65	852	3,422	108	810	2,778	114	689	2,084
P-value	0.144 ^a^			0.012 ^a^			0.812 ^a^		
Pre-pregnancy BMI (kg/m^2^)*									
< 18.5	15.4	14.1	8.0	18.3	14.0	8.9	19.6	10.2	4.4
18.5 - 24.9	78.9	77.4	74.0	75.0	72.7	70.7	69.1	71.0	64.7
25.0 - 29.9	5.8	7.8	15.1	4.8	12.0	16.2	10.3	14.6	20.9
≥ 30.0	0.0	0.7	2.9	1.9	1.4	4.2	1.0	4.3	10.0
**Total (N)**	52	696	2,920	104	786	2,698	97	630	1,918
P-value	< 0.001^a^			< 0.001^a^			< 0.001^a^		
	< 0.001 ^b^			< 0.001 ^b^			< 0.001 ^b^		
**Parity**									
0	92.3	78.9	40.0	96.2	72.1	34.5	95.6	75.8	38.8
1	7.7	18.4	33.1	3.8	22.8	32.7	2.6	18.9	29.5
2 or more	0.0	2.7	26.9	0.0	5.1	32.8	1.8	5.4	31.6
**Total (N)**	65	852	3,423	106	804	2,754	114	689	2,083
P-value		< 0.001^a^			< 0.001^a^			< 0.001^a^	
	< 0.001^b^			< 0.001 ^b^			< 0.001 ^b^		
**Marital status**									
Single	29.2	18.1	7.4	43.5	29.3	9.7	45.6	28.7	15.0
Married	70.8	81.9	92.6	56.5	70.7	90.3	54.4	71.3	85.0
**Total (N)**	65	852	3,421	108	810	2,779	114	689	2,084
P-value	< 0.001^a^			< 0.001^a^			< 0.001^a^		

Pelotas, Brazil, 1982, 1993, and 2004

^a ^Chi-square test

^b ^Chi-square test for trend

^* ^Variable with more missing values (n = 942 in 1982, n = 154 in 1993, and n = 368 in 2004)

Table 2 also shows how the characteristics of adolescent mothers evolved between 1982 and 2004. In this period, the proportion of adolescents who attained up to eight years of schooling rose by 20%, the proportion of adolescent mothers who were non-white increased by 37%, overweight prevalence rose by 87% and the proportion of adolescent mothers who were unmarried increased by almost 60%.

Potential mediating factors were also studied (see Additional file 1 - Table S1). Adolescent mothers were more likely to report fewer than six prenatal attendances, and gained less weight during pregnancy in relation to 20-29-year-old mothers. They were also less likely to present pregnancy complications (hypertension, diabetes or threatened abortion). Mothers aged 16-19 years were more likely to smoke during pregnancy and to have preterm or low birth weight babies, and less likely to have a C-section and to breastfeed for more than six months in comparison to mothers aged 20-29 years.

Perinatal and infant mortality rates were calculated for three groups of maternal age (< 16, 16-19 and 20-29 years) because there was sufficient number of deaths to analyze mothers younger than 16 years as a separate category (Table 3 and 4). For fetal, early neonatal, late neonatal and postneonatal mortality rates, it was only possible to stratify mortality rates by two maternal age categories (< 20 years and 20-29 years) (Table 5).

**Table 3:** Crude and adjusted ORs (95% CI) for perinatal mortality by maternal age

	1982			1993			2004				Pooled cohorts		
	
	Deaths/Births (PMR)*	Crude OR (95%CI)	Adjusted OR (95%CI) ¹	Deaths/Births (PMR)*	Crude OR (95%CI)	Adjusted OR (95%CI) ¹	Deaths/Births (PMR)*	Crude OR (95%CI)	Adjusted OR (95%CI) ¹	**P value**^**c**^	Deaths/Births (PMR)*	Crude OR (95%CI)^2^	Adjusted OR (95%CI) ^3^
**Maternal age, y**										0.859			
< 16	2/65 (30.8)	1.2 (0.4; 8.7)	1.9 (0.4; 8.7)	2/108 (18.5)	1.0 (0.2; 4.2)	0.8 (0.2; 3.4)	5/113 (44.2)	2.6 (1.0; 6.6)	1.7 (0.5; 5.0)		9/286 (31.5)	1.9 (1.0; 3.8)	1.4 (0.7; 2.9)
16-19	23/852 (27.0)	1.0 (0.6; 1.6)	1.1 (0.6; 2.2)	14/810 (17.3)	0.9 (0.5; 1.7)	0.8 (0.4; 1.5)	10/685 (14.6)	0.8 (0.4; 1.7)	0.7 (0.3; 1.5)		47/2,347 (20.0)	1.0 (0.7; 1.4)	0.8 (0.6; 1.2)
20-29	90/3,424 (26.3)	1.0	1.0	51/2,779 (18.4)	1.0	1.0	37/2,079 (17.8)	1.0	1.0		178/8,282 (21.5)	1.0	1.0
P-value		0.972 ^a^	0.707 ^a^		0.979 ^a^	0.752^a^		0.163 ^a^	0.265 ^a^			0.241 ^a^	0.373 ^a^
		0.838 ^b^	0.475 ^b^		0.885 ^b^	0.474 ^b^		0.376 ^b^	0.887 ^b^			0.313 ^b^	0.934 ^b^
**Maternal age, y**										0.725			
< 20	25/917 (27.3)	1.0 (0.7; 1.6)	1.2 (0.6; 2.3)	16/918 (17.4)	0.9 (0.9; 1.7)	0.8 (0.4; 1.5)	15/798 (18.8)	1.1 (0.6; 1.9)	0.8 (0.4; 1.7)		56/2,633 (21.3)	1.1 (0.8; 1.5)	0.9 (0.6; 1.3)
20-29	90/3,424 (23.4)	1.0	1.0	51/2,779 (18.4)	1.0	1.0	37/2,079 (17.8)	1.0	1.0		178/8,282 (21.5)	1.0	1.0
P-value		0.870 ^a^	0.599 ^a^		0.855 ^a^	0.451 ^a^		0.858 ^a^	0.545 ^a^			0.625 ^a^	0.540 ^a^

Pelotas, Brazil, 1982, 1993, and 2004

^* ^PMR (perinatal mortality rate) = number of perinatal deaths per 1,000 births (stillbirths and live births).

^a ^Likelihood ratio test.

^b ^Likelihood ratio test for trend.

^c ^Likelihood ratio test for interaction.

¹ Adjusted for family income, maternal schooling, maternal skin color, marital status, and parity.

^2 ^Adjusted for cohort year.

^3 ^Adjusted for variables in model ^1 ^plus model ^2^.

**Table 4:** Crude and adjusted ORs (95% CI) for infant mortality by maternal age

	1982			1993			2004				Pooled cohorts		
	
	Deaths/Births (IMR)*	Crude OR (95%CI)	Adjusted OR (95%CI) ¹	Deaths/Births (IMR)*	Crude OR (95%CI)	Adjusted OR (95%CI) ¹	Deaths/Births (IMR)*	Crude OR (95%CI)	Adjusted OR (95%CI) ¹	**P value**^**c**^	Deaths/Births (IMR)*	Crude OR (95%CI)^2^	Adjusted OR (95%CI) ^3^
**Maternal age, y**										0.563			
< 16	4/65 (61.5)	2.0 (0.7; 5.5)	2.1 (0.7; 6.3)	1/106 (9.4)	0.5 (0.1; 3.8)	0.8 (0.1; 6.6)	5/112 (44.6)	2.4 (0.9; 6.1)	2.3 (0.8; 6.5)		10/283 (35.3)	1.6 (0.9; 3.2)	1.9 (0.96; 3.9)
16-19	39/843 (46.3)	1.5 (1.0; 2.1)	1.6 (1.1; 2.5)	16/804 (19.9)	1.1 (0.6; 1.9)	1.4 (0.8; 2.7)	18/680 (26.5)	1.4 (0.8; 2.4)	1.5 (0.8; 2.8)		73/2,327 (31.4)	1.3 (1.0; 1.8)	1.5 (1.1; 2.1)
20-29	109/3,380 (32.2)	1.0	1.0	50/2,754 (18.2)	1.0	1.0	40/2,062 (19.4)	1.0	1.0		199/8,196 (24.3)	1.0	1.0
P-value		0.093 ^a^ 0.030 ^b^	0.059 ^a^ 0.019 ^b^		0.713 ^a^ 0.888 ^b^	0.522 ^a^ 0.449 ^b^		0.189 ^a^ 0.073 ^b^	0.259 ^a^ 0.101 ^b^			0.057 ^a^ 0.017 ^b^	0. 013 ^a^ 0.004 ^b^
**Maternal age, y**										0.463			
< 20	43/908 (47.4)	1.5 (1.0; 2.1)	1.7 (1.1; 2.5)	17/910 (18.7)	1.0 (0.6; 1.8)	1.4 (0.7; 2.6)	23/792 (29.0)	1.5 (0.9; 2.5)	1.6 (0.8; 2.9)		83/2,610 (31.8)	1.4 (1.1; 1.8)	1.6 (1.2; 2.1)
20-29	109/3,380 (32.2)	1.0	1.0	50/2,754 (18.2)	1.0	1.0	40/2,062 (19.4)	1.0	1.0		199/8,196 (24.3)	1.0	1.0
P-value		0.035 ^a^	0.020 ^a^		0.918 ^a^	0.318 ^a^		0.127 ^a^	0.154 ^a^			0.020 ^a^	0.004 ^a^
													

Pelotas, Brazil, 1982, 1993, and 2004

*IMR (infant mortality rate) = number of infant deaths per 1,000 live births.

^a ^Likelihood ratio test.

^b ^Likelihood ratio test for trend.

^c ^Likelihood ratio test for interaction.

¹ Adjusted for family income, maternal schooling, maternal skin color, marital status, and parity.

^2 ^Adjusted for cohort year.

^3 ^Adjusted for variables in model ^1 ^plus model ^2^.

**Table 5:** Crude and adjusted ORs (95% CI) for mortality outcomes by maternal age

	1982			1993			2004				Pooled cohorts		
Maternal age, y	Deaths/Births (MR)*	Crude OR (95%CI)	Adjusted OR (95%CI) ¹	Deaths/Births (MR)*	Crude OR (95%CI)	Adjusted OR (95%CI) ¹	Deaths/Births (MR)*	Crude OR (95%CI)	Adjusted OR (95%CI) ¹	**P value**^**b**^	Deaths/Births (MR)*	Crude OR (95%CI)^2^	Adjusted OR (95%CI) ^3^
**FETAL MORTALITY**													
										0.838			
< 20	9/917 (9.8)	0.8 (0.4; 1.6)	0.5 (0.3; 1.2)	8/918 (8.7)	1.0 (0.4; 2.2)	0.7 (0.3; 1.7)	6/803 (7.4)	0.9 (0.4; 2.3)	0.6 (0.2; 1.8)		23/2,638 (8.7)	0.9 (0.5; 1.4)	0.6 (0.4; 1.0)
20-29	44/3,424 (12.9)	1.0	1.0	25/2,779 (9.0)	1.0	1.0	17/2,084 (8.0)	1.0	1.0		86/8,287 (10.4)	1.0	1.0
P-value		0.446 ^a^	0.107 ^a^		0.937 ^a^	0.409 ^a^		0.852 ^a^	0.365 ^a^			0.522 ^a^	0.054 ^a^

**EARLY NEONATAL MORTALITY**													
										0.713			
< 20	16/908 (17.6)	1.3 (0.7; 2.3)	1.2 (0.6; 2.3)	8/910 (8.8)	0.9 (0.4; 2.1)	1.0 (0.4; 2.5)	9/792 (11.4)	1.2 (0.5; 2.6)	0.9 (0.3; 2.2)		33/2,610 (12.6)	1.2 (0.8; 1.7)	1.0 (0.7; 1.6)
20-29	46/3,380 (13.6)	1.0	1.0	26/2,754 (9.4)	1.0	1.0	20/2,062 (9.7)	1.0	1.0		92/8,196 (11.2)	1.0	1.0
P-value		0.380 ^a^	0.584 ^a^		0.859 ^a^	0.990 ^a^		0.695 ^a^	0.808 ^a^			0.479 ^a^	0.853 ^a^

**LATE NEONATAL MORTALITY**													
										0.656			
< 20	4/908 (4.4)	1.5 (0.5; 4.8)	1.1 (0.3; 3.9)	4/910 (4.4)	2.0 (0.6; 7.2)	1.9 (0.4; 7.8)	6/792 (7.6)	3.1 (1.0; 10.3)	2.5 (0.6; 10.5)		14/2,610 (5.4)	2.1 (1.1; 4.1)	1.6 (0.7; 3.4)
20-29	10/3,380 (3.0)	1.0	1.0	6/2,754 (2.2)	1.0	1.0	5/2,062 (2.4)	1.0	1.0		21/8,196 (2.6)	1.0	1.0
P-value		0.513 ^a^	0.901 ^a^		0.292 ^a^	0.399 ^a^		0.062 ^a^	0.216 ^a^			0.040 ^a^	0.272 ^a^

**POSTNEONATAL MORTALITY**													
										0.682			
< 20	23/908 (25.3)	1.6 (1.0; 2.7)	2.2 (1.3; 3.7)	5/910 (5.5)	0.8 (0.3; 2.3)	1.1 (0.3; 3.4)	8/792 (10.1)	1.4 (0.6; 3.3)	1.8 (0.6; 4.9)		36/2,610 (13.8)	1.4 (0.9; 2.1)	1.8 (1.1; 2.9)
20-29	53/3,380 (15.7)	1.0	1.0	18/2,754 (6.5)	1.0	1.0	15/2,062 (7.3)	1.0	1.0		86/8,196 (10.5)	1.0	1.0
P-value		0.061 ^a^	0.017 ^a^		0.726 ^a^	0.906 ^a^		0.460 ^a^	0.278 ^a^			0.100 ^a^	0.012 ^a^

Pelotas, Brazil, 1982, 1993, and 2004

*MR (mortality rate) = number of deaths per 1,000 live births for early neonatal, late neonatal, and postneonatal period. Fetal mortality rate is expressed as number of fetal deaths per 1,000 births (stillbirth and live birth).

^a ^Likelihood ratio test. ^b ^Likelihood ratio test for interaction.

¹ Adjusted for family income, maternal schooling, maternal skin color, marital status, and parity.

^2 ^Adjusted for cohort year.

^3 ^Adjusted for variables in model ^1 ^plus model ^2^.

The pooled perinatal mortality rates from the three cohorts were 31.5 deaths per 1,000 births for the adolescents younger than 16 years, 20.0 for those aged 16-19 years and 21.5 for mothers aged 20-29 years. Crude and adjusted ORs for perinatal mortality (Table 3) were somewhat higher for babies born to adolescents aged 12-15 years in comparison to 20-29-year-old mothers, but all confidence intervals included unity (Table 3).

The pooled infant mortality rates were 35.3 deaths per 1,000 births for adolescents younger than 16 years, 31.4 for adolescents aged 16-19 and 24.3 for 20-29-year-old mothers (Table 4). In the pooled crude analyses, infant mortality was inversely related to maternal age. The odds of infant death were 60% higher among 12-15-year-old mothers and 30% higher among 16-19-year-old mothers compared to 20-29-year-old mothers. After adjustment for confounders, these increased to 90% and 50% (Table 4), respectively, due to negative confounding by parity (see Additional file 2 - Table S2). Parous women - whether adolescents or 20-29-year-olds - had considerably worse socioeconomic indicators and higher mortality in their offspring, compared to those delivering their first child (see Additional file 3 - Table S3 and S4). As a consequence, the unadjusted analyses underestimated the strength of the association between adolescent pregnancies and infant mortality.

Further adjustment for mediating variables resulted in pooled ORs equal to 0.6 (95%CI = 0.2; 2.1) for mothers aged 12-15 years and 1.3 (95%CI = 0.9; 1.9) for mothers aged 16-19 years (see Additional file 4 - Table S5). Weight gain and antenatal care were the two mediating variables that mainly accounted for the change in odds ratios, whereas adjustment for type of delivery and birthweight did not lead to noticeable changes. When breastfeeding duration is added to this model, the odds ratio for infant mortality becomes equal to 1.0 (95% CI = 0.3; 3.0).

Fetal and early neonatal mortality did not show associations with maternal age. In the crude analyses, late neonatal mortality was higher among babies born to adolescent mothers in 2004 and also in the combined analyses, but the associations were attenuated after adjustment for confounders, and the confidence intervals included unity. Postneonatal mortality was associated with maternal age after controlling for confounders in 1982 and also in the pooled analyses. The odds of postneonatal death among infants born to adolescent mothers were 80% higher when compared to those born to 20-29-year-old mothers (Table 5). Further adjustment for mediating variables reduced the pooled ORs to 1.2 (95%CI = 0.7; 2.2) for mothers aged 12-19 years (see Additional file 4 - Table S5). As before, weight gain and antenatal care were the two mediating variables that mainly accounted for the change in odds ratios. When breastfeeding duration is added to this model, the odds ratio for postnatal mortality becomes equal to 1.0 (95% CI = 0.3; 3.0).

**Figure 1:**
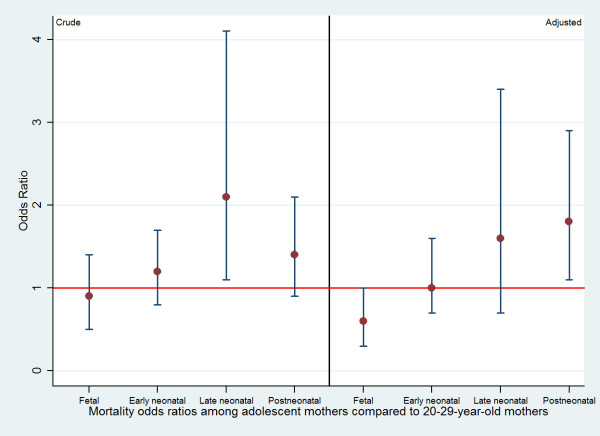
**Pooled crude and adjusted ORs for offspring mortality among 12-19-year-old mothers compared to 20-29-year-old mothers**. Adjusted for family income, maternal schooling, maternal skin color, marital status, parity and cohort year.

Figure 1 summarizes the pooled mortality ORs for fetal, neonatal, and postneonatal deaths, showing how the effects of adolescent childbearing seem to be more important for late than for early deaths.

## Discussion

We found an increased risk of infant mortality among children born to adolescent mothers compared to those who were born to mothers aged 20-29 years, after socioeconomic factors and parity were controlled for. This excess was due to postneonatal deaths. This association was attenuated when mediators were introduced into the model, suggesting that much of the excessive risk among infants from adolescent mothers is explained by behavioral and health care characteristics. Maternal age was not associated with perinatal mortality or either of its components - fetal or early neonatal deaths.

When comparing our results to the literature, it is important to consider which confounding variables were adjusted for in each study, as well as the characteristics of the populations where the studies were carried out. In terms of confounding factors, one should ideally include valid and multiple indicators of socioeconomic position (SEP), rather than rely on single indicators such as income, education, occupation or assets. Lack of thorough adjustment for SEP may lead to residual confounding given the strong association between poverty and adolescent childbearing. In addition, it is important to present analyses adjusted for mediating factors - such as antenatal care, weight gain during pregnancy, type of delivery, birth weight or breastfeeding - separately from those adjusted for confounding factors, as these models have different causal interpretations.

Two review articles are available in the literature. In 2001, Cunnington's systematic review identified four studies from high-income countries which assessed the effect of maternal age on neonatal mortality, of which two reported associations [13]. In 2009, a new review identified three papers on the same topic, of which one was included in the 2001 review [9]. All three papers reported increased odds of neonatal deaths among adolescent mothers when compared with mothers aged 20-29 years (from 1.1 to 2.7) [9]. All studies were carried out in high-income countries. Three out of four studies showing associations used educational level as a proxy for SEP and two of them also controlled for adequacy of prenatal care and for tobacco consumption [9,13]. No reviews of the effect of maternal age on infant mortality (rather than neonatal deaths) were located.

Through a systematic search of PubMed since the 1960's, we identified 22 studies reporting on maternal age and risk of fetal or infant mortality. In general, studies which failed to adjust for SEP tended to report that adolescent childbearing increased the risk of fetal [8,26,28], perinatal [18], neonatal [10,12,18,21,29], postneonatal [21,23,29,30], and infant mortality [12,16,37], whereas these outcomes were not associated to maternal age in analysis with adjustment for SEP [15,17,27,38]. Authors who adjusted for several socioeconomic variables reported ORs from 1.1 to 1.5 [15,17,27] and those who used just educational level as indicator of SEP or just ethnicity and marital status reported ORs from 1.6 to 3.0 [12,18,21,26,28-30]. Sixteen of the 22 studies were from high-income countries [8,10,12,15,16,18,19,21-23,26,28-30,37,38]. Comparing results of studies from high-income and those from low and middle-income countries, it appears that associations with maternal age tend to be stronger in the former for fetal mortality and neonatal mortality [8,10,12,18,21,26,28,29]. Differences between studies settings may be due to baseline risk of mortality, social characteristics of adolescents, type of health care system and social support available in each setting, among other factors. Methodological differences may also account for discrepancies in findings, including the fact that several studies from high-income countries relied on secondary databases with very large sample sizes but possibly lower data quality.

In addition, some studies [14,19] may have failed to detect associations between adolescent motherhood and offspring mortality because of adjustment for mediators such as birth weight, gestational age or medical and behavioral risk factors during pregnancy, which in fact may be a consequence of adolescent pregnancy rather than true confounding factors. Analyses adjusted for mediating factors are important but their interpretation is different from analyses adjusted for true confounders.

An interesting finding in our analyses was the increase in the effect of adolescent motherhood on infant mortality as child age increases from 1 to 12 months, which supports the social and environmental explanations for this relationship. If the excess of mortality among children of adolescent mothers is due to maternal physiological immaturity, then the effect of maternal age should be more pronounced for periods of the children closer to the time of birth, or equally pronounced across all child ages. Similar findings were described in other studies [17,21,30]. The fact that the association with post-natal mortality completely disappeared after adjustment for factors such as weight gain during pregnancy, antenatal care and breastfeeding is particularly important because it suggests potential areas for interventions to reduce mortality among offspring of pregnant adolescents.

Major strengths of this study are the population-based sample from birth cohort studies, the very high (over 99%) response rates at baseline, the detailed assessment of maternal characteristics, and active surveillance for fetal and infant deaths. In spite of the large sample sizes, deaths are rare events and some of our analyses - particularly for mothers aged 12-15 years - had low power, and this group had to be pooled with older adolescents, whose risk may be considerably lower. Further studies would be needed to replicate this analysis in a larger sample and in a similar setting.

## Conclusions

Offspring mortality is only one of several outcomes of adolescent childbearing. Our results suggest that socioeconomic and behavioral mechanisms may be more important than the biological effect of maternal age in predicting perinatal and infant survival. Therefore, given proper health care, economic and social support, the children of adolescents may have the same chances of surviving as those of older mothers. Nevertheless, there are other important social consequences of adolescent pregnancies [39], as well as consequences to their own health and growth [40], which strongly support interventions for delaying the age at childbearing.

## Competing interests

The authors declare that they have no competing interests.

## Authors' contributions

MCRM participated in the conception of the study, performed the statistical analysis, interpreted the results and drafted the manuscript. AJDB, ISS, AMBM, AM and FB participated in the acquisition of data and in the critical revision of the article. CGV participated in the design of the study, supervised the analysis and the interpretation of the findings, as well as the writing of the article. All authors read and approved the final manuscript.

## Pre-publication history

The pre-publication history for this paper can be accessed here:

http://www.biomedcentral.com/1471-2458/11/781/prepub

## Supplementary Material

Additional file 1 **Table S1 - Distribution of maternal reproductive health and offspring characteristics, by maternal age. Pelotas, Brazil, 1982, 1993, and 2004**. This table provides additional information regarding to maternal and offspring characteristics according to maternal age and birth cohort. Click here for file

Additional file 2 **Table S2 - Crude and adjusted ORs (95% CI) for infant mortality by maternal age. Pelotas, Brazil, 1982, 1993, and 2004**. Table S2 is similar to Table 4 in the published article. In Table 4, parity is also included as one of the confounding variables in the adjusted model, whereas in Table S2 it is not. Odds ratios in Table 4 are considerably larger than those in Table S2, showing that parity is a negative confounder in the association between adolescent childbearing and offspring mortality. Click here for file

Additional file 3 **Maternal age, parity and infant mortality**. Table S3 shows that women with previous children are considerably worse off than those delivering their first child and Table S4 shows that this strong association with poverty reverts the association between first birth and infant mortality. Click here for file

Additional file 4 **Table S5 - Adjusted ORs (95% CI) for postneonatal and infant mortality by maternal age after controlling for mediating variables. Pelotas, Brazil, 1982, 1993, and 2004**. This table shows the confounder-adjusted odds ratio for post-neonatal and for infant mortality becomes equal to 1.0 after adjustment for mediating factors - particularly weight gain during pregnancy, antenatal care and breastfeeding duration. Click here for file
