# Supplementary material for: Childbearing during adolescence and offspring mortality: findings from three population-based cohorts in southern Brazil
Source: BMC Public Health. 2011 Oct 10;11:781. doi: 10.1186/1471-2458-11-781 (PMC3207956; doi:10.1186/1471-2458-11-781)
Supplement: Additional file 1 — Table S1 - Distribution of maternal reproductive health and offspring characteristics, by maternal age. Pelotas, Brazil, 1982, 1993, and 2004. This table provides additional information regarding to maternal and offspring characteristics according to maternal age and birth cohort. [file 1471-2458-11-781-S1.DOC]

**Table S1 – Distribution of maternal reproductive health and offspring characteristics**, by maternal age. Pelotas, Brazil, 1982, 1993, and 2004

|  | **1982** | | |  | **1993** | | |  | **2004** | | |
| --- | --- | --- | --- | --- | --- | --- | --- | --- | --- | --- | --- |
| **Variables** | **<16** | **16-19** | **20-29** |  | **<16** | **16-19** | **20-29** |  | **<16** | **16-19** | **20-29** |
|  | **%** | **%** | **%** |  | **%** | **%** | **%** |  | **%** | **%** | **%** |
| **Maternal weight gain – Kg (tertile)^*^** |  |  |  |  |  |  |  |  |  |  |  |
| 1st (lower) | 37.5 | 38.1 | 31.6 |  | 33.0 | 40.0 | 34.0 |  | 21.4 | 28.6 | 27.5 |
| 2nd | 27.1 | 34.3 | 38.2 |  | 32.0 | 32.9 | 35.6 |  | 41.8 | 36.4 | 36.6 |
| 3rd (upper) | 35.4 | 27.7 | 30.1 |  | 35.0 | 27.1 | 30.5 |  | 36.7 | 35.0 | 36.0 |
| **Total (N)** | 48 | 607 | 2,602 |  | 100 | 778 | 2,650 |  | 98 | 632 | 1,934 |
| P-value | 0.022^a^ | | |  | 0.027^a^ | | |  | <0.643^a^ | | |
|  |  | | |  |  | | |  |  | | |
| **Number of prenatal care attendances** |  |  |  |  |  |  |  |  |  |  |  |
| Less than six | 55.4 | 48.1 | 32.2 |  | 42.6 | 34.7 | 25.3 |  | 38.7 | 29.1 | 16.8 |
| Six or more | 44.6 | 51.9 | 67.8 |  | 57.4 | 65.3 | 74.7 |  | 61.3 | 70.9 | 83.2 |
| **Total (N)** | 65 | 851 | 3,403 |  | 108 | 806 | 2,774 |  | 111 | 664 | 2,003 |
| P-value | <0.001 ^a^ | | |  | <0.001 ^a^ | | |  | <0.001 ^a^ | | |
|  |  | | |  |  | | |  |  | | |
| **Smoking during pregnancy** |  |  |  |  |  |  |  |  |  |  |  |
| No | 69.2 | 58.0 | 61.6 |  | 73.1 | 69.0 | 65.8 |  | 75.4 | 69.5 | 72.2 |
| Yes | 30.8 | 42.0 | 38.4 |  | 26.9 | 31.0 | 34.2 |  | 24.6 | 30.5 | 27.8 |
| **Total (N)** | 65 | 852 | 3,424 |  | 108 | 810 | 2,779 |  | 114 | 689 | 2,084 |
| P-value | 0.057 ^a^ | | |  | 0.081^a^ | | |  | 0.270 ^a^ | | |
|  |  | | |  |  | | |  |  | | |
| **Pregnancy complications** ^*^ |  |  |  |  |  |  |  |  |  |  |  |
| No | 86.2 | 91.3 | 87.3 |  | 80.2 | 75.1 | 72.4 |  | 73.7 | 70.6 | 67.1 |
| Yes | 13.9 | 8.7 | 12.7 |  | 19.8 | 24.9 | 27.6 |  | 26.3 | 29.4 | 32.9 |
| **Total (N)** | 65 | 852 | 3,421 |  | 106 | 799 | 2,760 |  | 114 | 688 | 2,081 |
| P-value |  | 0.005 ^a^ |  |  |  | 0.081^a^ |  |  |  | 0.102 ^a^ |  |
|  |  |  |  |  |  |  |  |  |  |  |  |
| **Type of delivery** |  |  |  |  |  |  |  |  |  |  |  |
| Vaginal | 66.1 | 78.6 | 74.9 |  | 70.4 | 75.8 | 72.2 |  | 65.8 | 66.5 | 57.2 |
| C-section | 33.9 | 21.36 | 25.1 |  | 29.6 | 24.2 | 27.8 |  | 34.2 | 33.5 | 42.8 |
| **Total (N)** | 65 | 852 | 3,424 |  | 108 | 810 | 2,779 |  | 114 | 689 | 2,084 |
| P-value |  | 0.016 ^a^ |  |  |  | 0.104 ^a^ |  |  | <0.001 ^a^ | | |
|  |  |  |  |  |  |  |  |  |  |  |  |
| **Preterm birth**^**^ |  |  |  |  |  |  |  |  |  |  |  |
| <37 weeks | 11.4 | 8.4 | 4.8 |  | 18.3 | 12.8 | 10.2 |  | 22.3 | 17.6 | 13.4 |
| 37+ weeks | 88.6 | 91.6 | 95.2 |  | 81.7 | 87.2 | 89.8 |  | 77.7 | 82.4 | 86.6 |
| **Total (N)** | 44 | 608 | 2,707 |  | 104 | 796 | 2,726 |  | 112 | 682 | 2,077 |
| P-value | 0.001 ^a^ | | |  |  | 0.007 ^a^ |  |  |  | 0.002 ^a^ |  |
|  |  |  |  |  |  |  |  |  |  |  |  |
| **Low birth weight** |  |  |  |  |  |  |  |  |  |  |  |
| <2500 g | 18.5 | 11.7 | 7.2 |  | 15.9 | 10.7 | 8.3 |  | 10.6 | 11.1 | 9.4 |
| 2500 + g | 81.5 | 88.3 | 92.7 |  | 84.1 | 89.3 | 91.7 |  | 89.4 | 88.9 | 90.6 |
| **Total** | 65 | 843 | 3,378 |  | 107 | 806 | 2,760 |  | 113 | 687 | 2,084 |
| P-value |  | <0.001^a^ |  |  |  | 0.006 ^a^ |  |  |  | 0.432 ^a^ |  |
|  |  |  |  |  |  |  |  |  |  |  |  |
| **Breastfeeding duration (months)** |  |  |  |  |  |  |  |  |  |  |  |
| Never | 10.5 | 9.1 | 6.7 |  | 3.2 | 8.1 | 5.0 |  | 3.8 | 2.4 | 3.0 |
| < 3.0 | 47.4 | 59.0 | 54.6 |  | 74.2 | 59.1 | 47.7 |  | 49.5 | 44.0 | 31.6 |
| 3.0 – 5.9 | 19.3 | 12.4 | 14.7 |  | 6.4 | 11.1 | 19.0 |  | 15.2 | 11.1 | 9.5 |
| 6.0 + | 22.8 | 19.5 | 24.0 |  | 16.1 | 21.7 | 28.3 |  | 37.1 | 42.5 | 50.1 |
| **Total** | 57 | 758 | 3,022 |  | 31 | 198 | 717 |  | 105 | 657 | 1,994 |
| P-value |  | <0.010^a^ |  |  |  | <0.001^a^ |  |  | <0.001^a^ | | |
|  |  | | |  |  | | |  |  | | |

^a^ Chi-square test

^*^ Variable with more missing values in 1993 (n=169) and in 2004 (n=223)

^**^ Variable with more missing values in 1982 (n=1,314)
