# Supplementary material for: Childbearing during adolescence and offspring mortality: findings from three population-based cohorts in southern Brazil
Source: BMC Public Health. 2011 Oct 10;11:781. doi: 10.1186/1471-2458-11-781 (PMC3207956; doi:10.1186/1471-2458-11-781)
Supplement: Additional file 2 — Table S2 - Crude and adjusted ORs (95% CI) for infant mortality by maternal age. Pelotas, Brazil, 1982, 1993, and 2004. Table S2 is similar to Table 4 in the published article. In Table 4, parity is also included as one of the confounding variables in the adjusted model, whereas in Table S2 it is not. Odds ratios in Table 4 are considerably larger than those in Table S2, showing that parity is a negative confounder in the association between adolescent childbearing and offspring mortality. [file 1471-2458-11-781-S2.DOC]

Table S2 - Crude and adjusted ORs (95% CI) for infant mortality by maternal age. Pelotas, Brazil, 1982, 1993, e 2004

|  | **1982** | | |  | **1993** | | |  | **2004** | | |  | **Pooled cohorts** | | |
| --- | --- | --- | --- | --- | --- | --- | --- | --- | --- | --- | --- | --- | --- | --- | --- |
|  | **Deaths/**  **Births**  **(IMR)^*^** | **Crude OR (95%CI)** | **Adjusted OR (95%CI) ¹** |  | **Deaths/**  **Births**  **(IMR)^*^** | **Crude OR (95%CI)** | **Adjusted OR (95%CI) ¹** |  | **Deaths/**  **Births**  **(IMR)^*^** | **Crude OR (95%CI)** | **Adjusted OR (95%CI) ¹** | ***P* ^c^** | **Deaths/**  **Births**  **(IMR)^*^** | **Crude OR (95%CI)²** | **Adjusted OR (95%CI) ³** |
| **Maternal age, y** |  |  |  |  |  |  |  |  |  |  |  | 0.587 |  |  |  |
| <16 | 4/65  (61.5) | 2.0  (0.7; 5.5) | 1.2  (0.4; 3.5) |  | 1/106  (9.4) | 0.5  (0.1; 3.8) | 0.4  (0.1; 3.1) |  | 5/112  (44.6) | 2.4  (0.9; 6.1) | 1.7  (0.6; 4.7) |  | 10/283  (35.3) | 1.6  (0.9; 3.2) | 1.1  (0.6; 2.2) |
| 16-19 | 39/843  (46.3) | 1.5  (1.0; 2.1) | 1.1  (0.7; 1.6) |  | 16/804  (19.9) | 1.1  (0.6; 1.9) | 1.0  (0.6; 1.8) |  | 18/680  (26.5) | 1.4  (0.8; 2.4) | 1.2  (0.7; 2.2) |  | 73/2,327  (31.4) | 1.3  (1.0; 1.8) | 1.1  (0.8; 1.4) |
| 20-29 | 109/3,380  (32.2) | 1.0 | 1.0 |  | 50/2,754  (18.2) | 1.0 | 1.0 |  | 40/2,062  (19.4) | 1.0 | 1.0 |  | 199/8,196  (24.3) | 1.0 | 1.0 |
| P-value |  | 0.093 ^a^  0.030 ^b^ | 0.881 ^a^  0.622 ^b^ |  |  | 0.713 ^a^  0.888 ^b^ | 0.623 ^a^  0.596 ^b^ |  |  | 0.189 ^a^  0.073 ^b^ | 0.546 ^a^  0.283 ^b^ |  |  | 0.057 ^a^  0.017 ^b^ | 0. 850 ^a^  0.569 ^b^ |
| **Maternal age, y** |  |  |  |  |  |  |  |  |  |  |  | 0.885 |  |  |  |
| <20 | 43/908  (47.4) | 1.5  (1.0; 2.1) | 1.1  (0.7; 1.6) |  | 17/910  (18.7) | 1.0  (0.6; 1.8) | 0.9  (0.5; 1.7) |  | 23/792  (29.0) | 1.5  (0.9; 2.5) | 1.3  (0.7; 2.2) |  | 83/2,610  (31.8) | 1.4  (1.1; 1.8) | 1.1  (0.8; 1.4) |
| 20-29 | 109/3,380  (32.2) | 1.0 | 1.0 |  | 50/2,754  (18.2) | 1.0 | 1.0 |  | 40/2,062  (19.4) | 1.0 | 1.0 |  | 199/8,196  (24.3) | 1.0 | 1.0 |
| P-value |  | 0.035 ^a^ | 0.661 ^a^ |  |  | 0.918 ^a^ | 0.798 ^a^ |  |  | 0.127 ^a^ | 0.378 ^a^ |  |  | 0.020 ^a^ | 0.580 ^a^ |
|  |  |  |  |  |  |  |  |  |  |  |  |  |  |  |  |

^*^ IMR (infant mortality rate) = number of infant deaths per 1 000 live births.

^a^ Likelihood ratio test.

^b^ Likelihood ratio test for trend.

^c^ Likelihood ratio test for interaction.

¹ Adjusted for family income, maternal schooling, maternal skin color, and marital status **(model without adjustment for parity).**

² Adjusted for cohort year.

³ Adjusted for variables in model ¹ plus model ².

**Note:** Table S2 is similar to Table 4 in the published article. In Table 4, parity is also included as one of the confounding variables in the adjusted model, whereas in Table S2 it is not. Odds ratios in Table 4 are considerably larger than those in Table S2, showing that parity is a negative confounder in the association between adolescent childbearing and offspring mortality.
