# Supplementary material for: Childbearing during adolescence and offspring mortality: findings from three population-based cohorts in southern Brazil
Source: BMC Public Health. 2011 Oct 10;11:781. doi: 10.1186/1471-2458-11-781 (PMC3207956; doi:10.1186/1471-2458-11-781)
Supplement: Additional file 3 — Maternal age, parity and infant mortality. Table S3 shows that women with previous children are considerably worse off than those delivering their first child and Table S4 shows that this strong association with poverty reverts the association between first birth and infant mortality. [file 1471-2458-11-781-S3.DOC]

Table S3. Description of maternal characteristics according to maternal age and parity

|  | **Pooled % (95% CI)** | | | |
| --- | --- | --- | --- | --- |
|  | **12 – 19** | | **20 – 29** | |
|  | **Nulliparous** | **Parous** | **Nulliparous** | **Parous** |
| Family income ≤ 1.0 mw^*^ | 29.4 (27.4; 31.4) | 35.5 (31.6; 39.4) | 15.1 (13.8; 16.3) | 23.4 (22.2; 24.5) |
| Schooling ≤ 4 years | 24.6 (22.7; 26.5) | 44.0 (40.0; 48.0) | 15.1 (13.8; 16.3) | 31.0 (29.8; 32.3) |
| Black or mixed | 23.1 (21.3; 24.9) | 30.4 (26.6; 34.1) | 16.7 (15.4; 18.0) | 24.4 (23.2; 25.5) |
| Single | 29.6 (27.6; 31.5) | 17.1 (14.0; 20.1) | 13.9 (12.7; 15.1) | 7.7 (7.0; 8.4) |
| Total | 2,052 | 586 | 3,238 | 5,147 |

**^*^** Minimum wages

Table S4. Infant mortality rates (IMR) according to maternal age and parity

|  | **IMR per 1000 (95% CI)** | | | |
| --- | --- | --- | --- | --- |
| **Maternal age, y** | **N** | **Nulliparous** | **N** | **Parous** |
| 12 – 19 | 2,052 | 27.6 (20.5; 34.8) | 3,138 | 46.3 (29.2; 63.4) |
| 20 – 29 | 3,138 | 19.0 (14.2; 23.8) | 5,147 | 27.5 (23.0; 32.0) |
