# Supplementary material for: Childbearing during adolescence and offspring mortality: findings from three population-based cohorts in southern Brazil
Source: BMC Public Health. 2011 Oct 10;11:781. doi: 10.1186/1471-2458-11-781 (PMC3207956; doi:10.1186/1471-2458-11-781)
Supplement: Additional file 4 — Table S5 - Adjusted ORs (95% CI) for postneonatal and infant mortality by maternal age after controlling for mediating variables. Pelotas, Brazil, 1982, 1993, and 2004. This table shows the confounder-adjusted odds ratio for post-neonatal and for infant mortality becomes equal to 1.0 after adjustment for mediating factors - particularly weight gain during pregnancy, antenatal care and breastfeeding duration. [file 1471-2458-11-781-S4.DOC]

Table S5 – Pooled adjusted ORs (95% CI) for postneonatal and infant mortality by maternal age after controlling for mediating variables. Pelotas, Brazil, 1982, 1993, and 2004

|  | **Posneonatal mortality** | | |  | **Infant mortality** | | |
| --- | --- | --- | --- | --- | --- | --- | --- |
|  | **Adjusted OR (95%CI)¹** | **Adjusted OR (95%CI)^2^** | **Adjusted OR (95%CI)^3^** |  | **Adjusted OR (95%CI)¹** | **Adjusted OR (95%CI)^2^** | **Adjusted OR (95%CI)^3^** |
| **Maternal age, y** |  |  |  |  |  |  |  |
| <16 | 0.6  (0.1; 4.6) | 0.7  (0.1; 5.4) | --  (--; --) |  | 0.6  (0.2; 2.1) | 0.5  (0.1; 2.2) | --  (--; --) |
| 16-19 | 1.3  (0.7; 2.3) | 1.3  (0.7; 2.5) | 1.0  (0.3; 3.2) |  | 1.3  (0.9; 1.9) | 1.3  (0.8; 2.0) | 1.1  (0.4; 3.2) |
| 20-29 | 1.0 | 1.0 | 1.0 |  | 1.0 | 1.0 | 1.0 |
| P-value | 0.577 ^a^  0.708 ^b^ | 0.677 ^a^  0.715 ^b^ | 0. 945 ^a^  0.824 ^b^ |  | 0.191 ^a^  0.462 ^b^ | 0.289 ^a^  0.697 ^b^ | 0.852 ^a^  0.913 ^b^ |
| **Maternal age, y** |  |  |  |  |  |  |  |
| <20 | 1.2  (0.7; 2.2) | 1.2  (0.6; 2.3) | 1.0  (0.3; 3.0) |  | 1.3  (0.9; 1.8) | 1.2  (0.8; 1.9) | 1.0  (0.3; 3.0) |
| 20-29 | 1.0 | 1.0 | 1.0 |  | 1.0 | 1.0 | 1.0 |
| P-value | 0.504 ^a^ | 0.569 ^a^ | 0.963 ^a^ |  | 0.211 ^a^ | 0.377 ^a^ | 0.946 ^a^ |
|  |  |  |  |  |  |  |  |

^a^ Likelihood ratio test.

^b^ Likelihood ratio test for trend.

¹ Adjusted for confounding factors plus weight gain, smoking, number of prenatal care, risk conditions during pregnancy and type of delivery.

^2^ Adjusted for model ^1^ plus gestational age and offspring birth weight.

^3^ Adjusted for model ^2^ plus breastfeeding duration.
